# Enhancement of Perovskite Solar Cells Efficiency using N-Doped TiO_2_ Nanorod Arrays as Electron Transfer Layer

**DOI:** 10.1186/s11671-016-1811-0

**Published:** 2017-01-17

**Authors:** Zhen-Long Zhang, Jun-Feng Li, Xiao-Li Wang, Jian-Qiang Qin, Wen-Jia Shi, Yue-Feng Liu, Hui-Ping Gao, Yan-Li Mao

**Affiliations:** 1 School of Physics and Electronics, Henan University, Kaifeng, 475004 China; 2 Institute for Computational Materials Science, Henan University, Kaifeng, 475004 China

**Keywords:** Enhancement of efficiency, N-doped TiO_2_ nanorod arrays, Electron transfer layer

## Abstract

In this paper, N-doped TiO_2_ (N-TiO_2_) nanorod arrays were synthesized with hydrothermal method, and perovskite solar cells were fabricated using them as electron transfer layer. The solar cell performance was optimized by changing the N doping contents. The power conversion efficiency of solar cells based on N-TiO_2_ with the N doping content of 1% (N/Ti, atomic ratio) has been achieved 11.1%, which was 14.7% higher than that of solar cells based on un-doped TiO_2_. To get an insight into the improvement, some investigations were performed. The structure was examined with X-ray powder diffraction (XRD), and morphology was examined by scanning electron microscopy (SEM). Energy dispersive spectrometer (EDS) and Tauc plot spectra indicated the incorporation of N in TiO_2_ nanorods. Absorption spectra showed higher absorption of visible light for N-TiO_2_ than un-doped TiO_2_. The N doping reduced the energy band gap from 3.03 to 2.74 eV. The photoluminescence (PL) and time-resolved photoluminescence (TRPL) spectra displayed the faster electron transfer from perovskite layer to N-TiO_2_ than to un-doped TiO_2_. Electrochemical impedance spectroscopy (EIS) showed the smaller resistance of device based on N-TiO_2_ than that on un-doped TiO_2_.

**Electronic supplementary material:**

The online version of this article (doi:10.1186/s11671-016-1811-0) contains supplementary material, which is available to authorized users.

## Background

In recent years, extensive studies are focused on perovskite solar cells (PSCs) due to their outstanding properties, such as large absorption coefficient, electron-hole diffusion length, and high charge carrier mobility [1–5]. The power conversion efficiency (PCE) of perovskite solar cells has been over 22% [6]. Conventionally, perovskite solar cells consist of a perovskite layer sandwiched between an electron transfer material (ETM) layer and a hole transfer material (HTM) layer. Mesoporous TiO_2_ has been used as the ETM in most of the perovskite solar cells [7, 8]. Compared with the mesoporous structure, one dimensional (1D) nanostructure has some advantages, such as easy pore filling of active layer or HTM, better electron transfer, and lower charge recombination [9, 10]. Therefore, TiO_2_ nanorods (NRs) have been widely applied to perovskite solar cells [11, 12]. However, there are a mass of oxygen vacancies defects exist in pristine TiO_2_ nanorods, which reduces the efficiency and stability of the perovskite solar cell [13].

In order to solve the issues, some methods have been adopted, such as metal doping [14, 15] and nonmetal doping [16]. It has been reported that N-doped TiO_2_ as a photoanode of dye-sensitized solar cells (DSSCs) can improve the energy conversion efficiency due to the change of properties of TiO_2_, such as electron lifetime prolongation, charge transfer resistance reduction, and visible light absorption extension [17, 18].

We wondered about the effect of N-doped TiO_2_ on the performance of perovskite solar cells. Hence, in the present study, we synthesized N-doped TiO_2_ (N-TiO_2_) nanorod arrays with hydrothermal method and fabricated perovskite solar cells using them as electron transfer layer. The solar cell performance was optimized by changing the N doping contents. The PCE of solar cells based on N-TiO_2_ with the N doping content of 1% (N/Ti, atomic ratio) has been achieved 11.1%, which was 14.7% higher than that of solar cells based on un-doped TiO_2_. The possible mechanisms of enhancement were discussed based on some investigations.

## Methods

### Growth of TiO_2_ Nanorod Arrays

Patterned fluorine-doped tin oxide (FTO)-coated glass substrate was cleaned by sonication for 20 min in detergent, acetone, 2-propanol, and ethanol, respectively. A TiO_2_ compact layer was deposited by dipping the substrate in a 0.2 M TiCl_4_ aqueous solution at 70 °C for 30 min. TiO_2_ NRs were grown on the treated FTO substrate by a hydrothermal method in our previous report [19]. A 0.7 mL of titanium(IV) n-butoxide was added to a mixture of hydrochloric acid and deionized water. Subsequently, the pre-calculated amount of CO(NH_2_)_2_ was added to the solution (the nominal N/Ti atomic ratio, 0.5, 1, 2, and 3%) and stirred until it was completely dissolved. The FTO substrate was put into the solution and sealed in an autoclave. The autoclave was heated to 170 °C for several hours. The obtained TiO_2_ nanorods film was rinsed and annealed at 500 °C for 60 min.

### Materials Preparation

Methylammonium iodide (CH_3_NH_3_I) was synthesized with a reported method [20]. The precursor solution for perovskite film formation was obtained by mixing PbCl_2_ and CH_3_NH_3_I in anhydrous *N*,*N*-dimethylformamide (DMF) at a 1:3 molar ratio at 60 °C overnight.

### Solar Cell Fabrication

The perovskite film was formed by spin coating at 2000 rpm for 60 s in a glove box, and drying on a hotplate at 110 °C for 60 min. The HTM layer was obtained by spin coating a spiro-OMeTAD solution at 2000 rpm for 60 s. Finally, a gold layer was deposited on the top of the device by thermal evaporation.

### Characterization

X-ray diffraction (XRD) patterns were measured using a diffractometer (DX-2700). Photocurrent–voltage (*I*–*V*) curves were carried out with a Keithley 2440 Source meter under AM 1.5 G illumination from a Newport Oriel Solar Simulator with an intensity of 100 mW/cm^2^. A shadow mask was used to determine the active area of 0.1 cm^2^. Morphologies and microstructures were performed using a scanning electron microscope (SEM, JEM-7001F, JEOL) equipped with an energy dispersive spectrometer (EDS). Absorption spectra were obtained with a UV–Vis spectrophotometer (Varian Cary 5000). Steady-state photoluminescence (PL) and time-resolved photoluminescence (TRPL) spectra were collected with a fluorometer (FLS 980E, Edinburgh Photonics). An electrochemical workstation (CHI660e, Shanghai CHI Co., Ltd.) was used to collect the electrochemical impedance spectroscopy (EIS) with a bias of 0.6 V.

## Results and Discussion

**Fig. 1:**
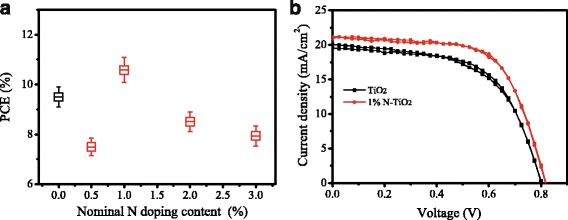
**a** PCE of solar cells dependence on nominal N doping contents. The data were obtained from 20 pieces of devices for each of conditions. **b** *I*–*V* curves of best performance solar cells based on un-doped TiO_2_ and 1% N-TiO_2_ NRs

TiO_2_ nanorod arrays with different N doping contents were prepared, and perovskite solar cells based on them were fabricated. The *I*–*V* measurements were performed by reverse scan (RS) and forward scan (FS). The photovoltaic parameters were obtained by the average of reverse scan and forward scan for each device. The *I*_sc_, *V*_oc_, FF, and PCE of the solar cells in the study were obtained by an average of the data from 20 pieces of devices. Figure 1a shows the power conversion efficiency of solar cells dependence on nominal N doping contents. The PCE changes with the increase of N doping content, which reaches the maximum at the doping content of 1% (N/Ti, atomic ratio). Table 1 shows the photovoltaic parameters of the solar cells based on un-doped and 1% N-TiO_2_ NRs. The *I*_sc_, *V*_oc_, FF, and PCE of the solar cells based on N-TiO_2_ NRs are enhanced compared with those based on un-doped TiO_2_ NRs. The PCE of solar cells based on N-TiO_2_ NRs is 14.7% higher than that of solar cells based on un-doped TiO_2_ NRs. Figure 1b shows the *I*–*V* curves of best performance solar cells based on un-doped TiO_2_ and 1% N-TiO_2_ NRs.

**Table 1:** Photovoltaic parameters of best performance solar cells based on the TiO_2_ and N-TiO_2_ NRs

Sample	*V*_oc_ (V)	*I*_sc_ (mA/cm^2^)	FF	PCE (%)
TiO_2_ 1% N-TiO_2_	0.80 ± 0.02 0.82 ± 0.01	19.2 ± 0.0.6 20.5 ± 0.7	0.62 ± 0.03 0.65 ± 0.02	9.5 ± 0.3 10.9 ± 0.2

**Fig. 2:**
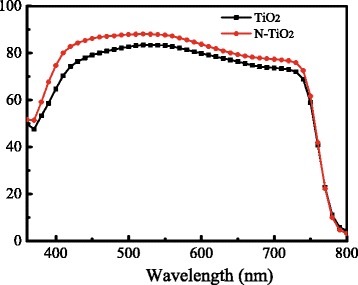
IPCE spectra of the perovskite solar cells based on un-doped TiO_2_ and 1% N-TiO_2_ NRs

Figure 2 shows the IPCE of the perovskite solar cells based on un-doped TiO_2_ NRs and 1% N-TiO_2_ NRs. The IPCE at the entire wavelength range of the cells based on N-TiO_2_ NRs are larger than those on the un-doped TiO_2_ NRs, which agree with the *I*–*V* measurements.

**Fig. 3:**
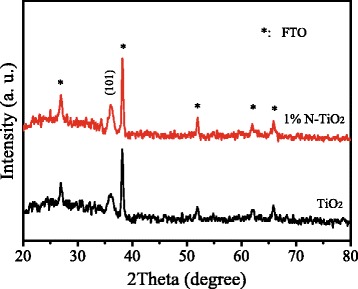
XRD patterns of un-doped TiO2 and 1% N-TiO2 NRs

To get an insight into the enhancement, some investigations were performed. Figure 3 shows the XRD patterns of un-doped TiO_2_ and 1% N-TiO_2_ NRs. The peaks labeled with stars were assigned to SnO_2_ (JCPDS card, 41-1445) on FTO substrate. The peak at 36.1° was assigned to the (101) planes of rutile TiO_2_ (JCPDS card, 21-1276) [21]. For the XRD patterns of N-TiO_2_ NRs, the peaks on N element were not observed. This could be due to the homogeneous distribution of N with Ti in the samples [22] and small amount of doping contents.

**Fig. 4:**
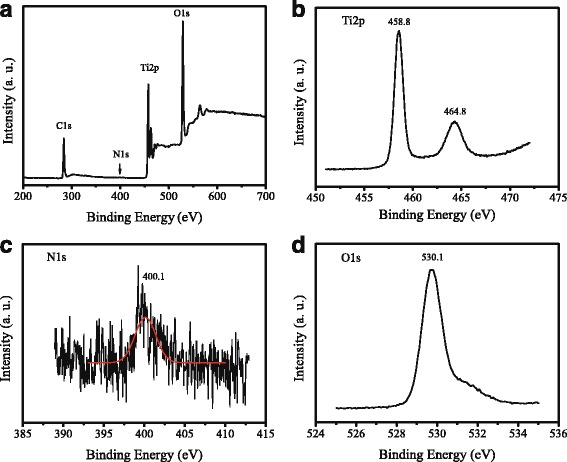
XPS spectra of 1% N-TiO_2_ NRs. **a** Survey XPS, **b** Ti 2p, **c** N 1s, and **d** O 1s

Figure 4 shows the XPS spectra of 1% N-TiO_2_ NRs. Figure 4a shows the survey XPS spectrum. The peaks at 458.8 and 464.8 eV in Fig. 4b are attributed to the binding energy of Ti 2p_3/2_ and Ti 2p_1/2_, respectively. The peak located at 400.1 eV in Fig. 4c is attributed to N 1s [23]. The peak at 530.1 eV in Fig. 4d could be from O 1s. The XPS spectra demonstrated the successful doping of N in the TiO_2_ film, which was further confirmed by the EDS spectrum as shown in Additional file 1: Figure S1 (Supplementary Material).

**Fig. 5:**
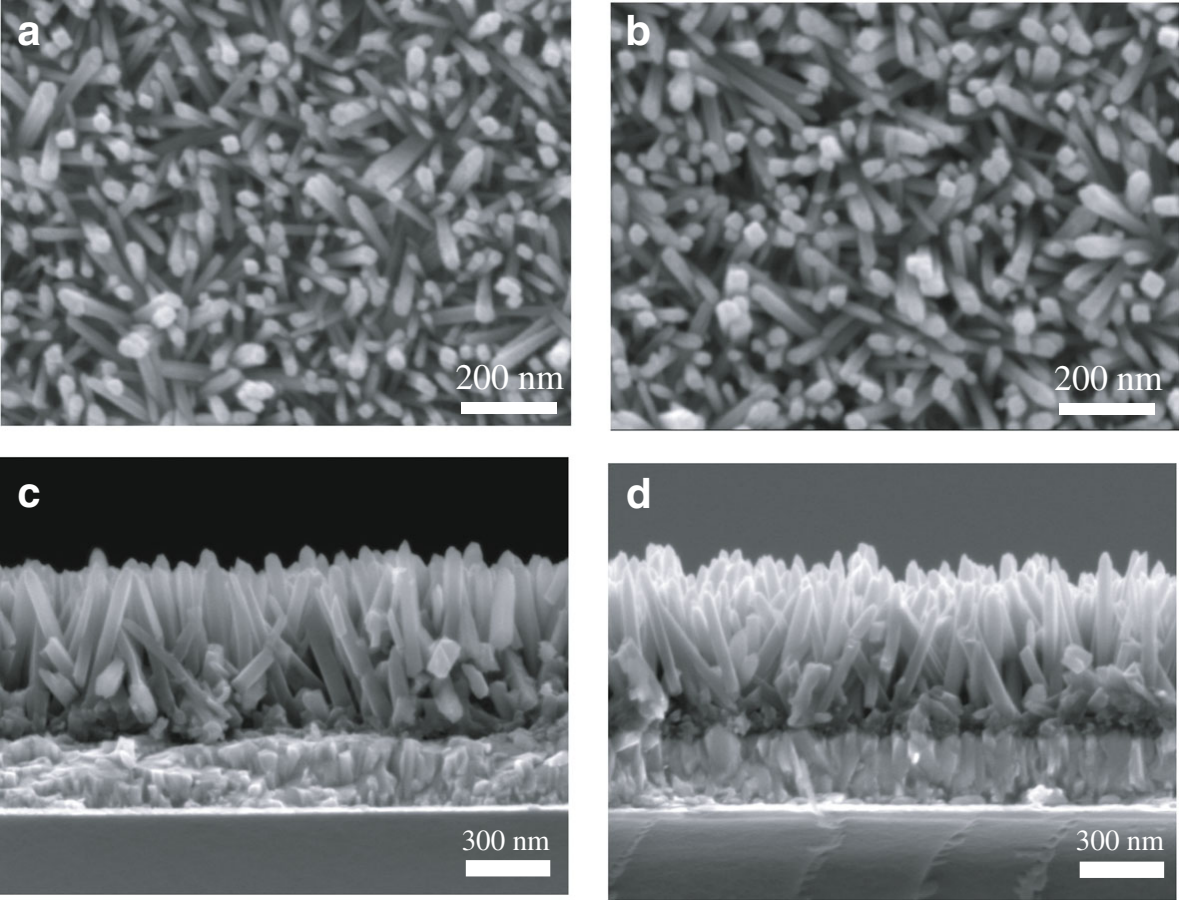
Plane-view SEM images of un-doped TiO_2_ (**a**) and 1% N-TiO_2_ NRs (**b**). Cross-sectional SEM images of un-doped TiO_2_ (**c**) and 1% N-TiO_2_ NRs (**d**)

Figure 5a, c shows the plane-view and cross-sectional SEM images of un-doped TiO_2_ NRs, and Fig. 5b, d shows the plane-view and cross-sectional SEM images of 1% N-TiO_2_ NRs. The diameter and length of the un-doped TiO_2_ NRs were determined to be 48 ± 5 nm and 490 ± 15 nm, respectively. The diameter and length of the 1% N-TiO_2_ NRs were determined to be 42 ± 6 nm and 480 ± 25 nm, respectively. The diameter of N-TiO_2_ NRs is slightly decreased compared with that of un-doped TiO_2_ NRs. The length distribution of N-TiO_2_ NRs is more ununiform than that of un-doped TiO_2_ NRs. From a view of large area (Additional file 1: Figure S2, Supplementary Material), there are some bluges on the sample surfaces due to the inhomogenous oritiation. There are more bluges on the surfaces of N-TiO_2_ NRs compared with those of un-doped TiO_2_ NRs. This could be attributed to the effect of N doping. Additional file 1: Figure S3 (Supplementary Material) shows the cross-sectional SEM images of the whole solar cells based on TiO_2_ NRs. The bottom is the FTO glass. Pores of nanorods are filled by MAPbI_3−*x*_Cl_*x*_, on which a capping layer of perovskite was formed. The Spiro-OMeTAD layer is separated by the capping layer from nanorod films. The top layer is a thin Au film.

**Fig. 6:**
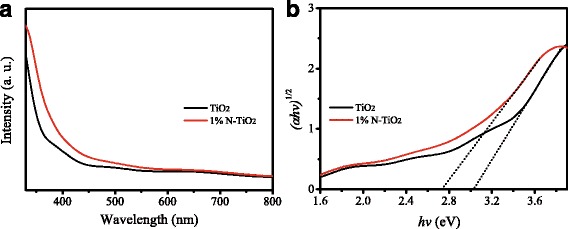
**a** UV–Vis absorption spectra of un-doped TiO_2_ and 1% N-TiO_2_ NRs. **b** Tauc curves

Figure 6a shows the UV–Vis absorption spectra of un-doped TiO_2_ and 1% N-TiO_2_ NRs. The absorption intensity of N-TiO_2_ is stronger than that of un-doped TiO_2_. The energy band gap (Eg) can be calculated based on the absorption spectra using the Tauc equation [24]. The Tauc curve is shown in Fig. 6b, in which Eg can be estimated to be 3.03 and 2.74 eV for un-doped TiO_2_ and N-TiO_2_ NRs, respectively. Compared with that of un-doped TiO_2_, the energy band gap of N-TiO_2_ becomes smaller. This could be attributed to the substitution location of N in the TiO_2_ lattice, in which an O (Ti) atom is replaced by an N atom [25]. The conduction band offset between N-TiO_2_ and MAPbI_3−*x*_Cl_*x*_ is larger than that between un-doped TiO_2_ and MAPbI_3−*x*_Cl_*x*_ due to its narrow energy band gap, which might be one of the reasons to present a higher voltage for N-TiO_2_-based solar cells [26].

**Fig. 7:**
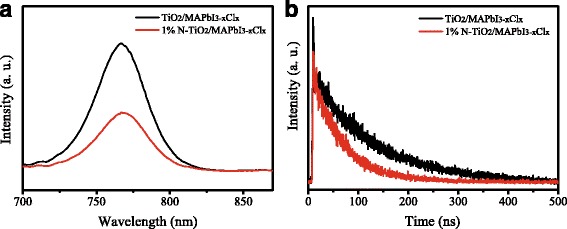
**a** PL and **b** TRPL spectra of un-doped TiO_2_/MAPbI_3−*x*_Cl_*x*_ and 1% N-TiO_2_/MAPbI_3−*x*_Cl_*x*_

PL is a suitable tool to study the efficiency of charge carrier trapping, migration, and transfer [27, 28]. Figure 7a shows the PL spectra of un-doped TiO_2_/MAPbI_3−*x*_Cl_*x*_ and 1% N-TiO_2_/MAPbI_3−*x*_Cl_*x*_. The intensity of PL spectrum was decreased due to the electron transfer when the MAPbI_3−*x*_Cl_*x*_ film contacts with TiO_2_ nanorod layer. The intensity decrease for N-TiO_2_ is more significant than that for un-doped TiO_2_, which indicates the electron transfer from MAPbI_3−*x*_Cl_*x*_ layer to N-TiO_2_ is more effective than to un-doped TiO_2_.

Figure 7b shows the TRPL spectra of un-doped TiO_2_/MAPbI_3−*x*_Cl_*x*_ and 1% N-TiO_2_/MAPbI_3−*x*_Cl_*x*_. The TRPL curves were analyzed with a bi-exponential decay function containing a fast decay (τ_1_) component and a slow decay component (τ_2_), and the detailed parameters are listed in Table 2. The fast decay component would be the result of free carrier transportation from MAPbI_3−*x*_Cl_*x*_ to TiO_2_, and the slow decay component could be the result of radiative decay [29, 30]. In the case of un-doped TiO_2_/MAPbI_3−*x*_Cl_*x*_, the fast decay time is 55.1 ns, and the slow decay time is 121.4 ns, while their weight fractions are 31.3 and 67.7%, respectively. Compared with those of un-doped TiO_2_/MAPbI_3−*x*_Cl_*x*_, the fast decay lifetime of N-TiO_2_/MAPbI_3−*x*_Cl_*x*_ is decreased to 36.4 from 55.1 ns, and the slow decay life time to 109.5 from 121.4 ns, while the weight fraction of fast decay is increased to 35.1 from 31.3%. This suggests that N-TiO_2_/MAPbI_3−*x*_Cl_*x*_ interface presents a faster charge transfer and induced charge recombination than the un-doped TiO_2_/MAPbI_3−*x*_Cl_*x*_ interface. The performance difference between the solar cells based on un-doped TiO_2_ and N-doped TiO_2_ NRs could be due to the property change of ETM, which affects the charge behavior in the interfaces [31, 32].

**Table 2:** Parameters of the TRPL spectra

Sample	τ_1_/ns	% of τ_1_	τ_2_/ns	% of τ_2_
TiO_2_ /MAPbI_3−*x*_Cl_*x*_	55.1	31.3	121.4	67.7
1% N-TiO_2_ /MAPbI_3−*x*_Cl_*x*_	36.4	35.1	109.5	64.9

**Fig. 8:**
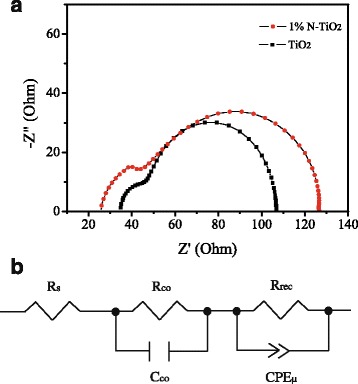
**a** EIS spectra of solar cells that based on un-doped TiO_2_ and 1% N-TiO_2_ NRs based solar cells. **b** Equivalent circuit for fitting the EIS data

To understand the charge transfer behavior of the solar cells, electrochemical impedance spectrum (EIS) was measured. Figure 8a shows the Nyquist plots of solar cells that based on un-doped TiO_2_ and 1% N-TiO_2_ NRs. The EIS contains two RC arcs. The arc at high frequency is originated from the contact resistance of the interfaces and that at low frequency is attributed to recombination resistance (*R*_rec_) and chemical capacitance (*C*_μ_) of the device [33, 34]. Figure 8b shows an equivalent circuit which was applied to fit the EIS. Table 3 lists the fitting parameters. The solar cells based on N-TiO_2_ present smaller series resistance and larger recombination resistance than those on un-doped TiO_2_. This demonstrates that the charge transport ability was enhanced and the carrier recombination rate was induced for the device on N-TiO_2_. There are many surface and bulk trap states due to oxygen vacancies for TiO_2_. It demonstrated that the incorporation of N atom into the lattice of TiO_2_ can decrease the traps density probably due to oxygen vacancy reduction [18, 35]. The smaller series resistance of N-TiO_2_ NRs could be due to the decreased traps density. The larger recombination resistance of N-TiO_2_ NRs may be contributed to the inactive N element leading to the increase of surface resistance [36, 37].

**Table 3:** Fitting parameters of EIS date

Sample	*R*_s_/Ω	*R*_co_/Ω	*R*_rec_/Ω	CPE-T/F
TiO_2_	34.8	59.3	12.7	6.3 × 10^−6^
1% N-TiO_2_	27.5	23.7	75.1	5.9 × 10^−6^

## Conclusions

In the present study, N-TiO_2_ NRs were synthesized with hydrothermal method, and perovskite solar cells based on them were fabricated. Compared with the solar cells based on un-doped TiO_2_, solar cells based on N-TiO_2_ present an enhanced performance. The solar cell performance was optimized by changing the N doping contents. The PCE of solar cells based on N-TiO_2_ with the N doping content of 1% (N/Ti, atomic ratio) has been achieved 11.1%, which was 14.7% higher than that of un-doped TiO_2_-based solar cells. To explain this phenomenon, some investigations were performed. The results indicate that the larger V_oc_ could be due to the larger conduction band offset resulting from the smaller energy band gap for N-TiO_2_, and the enlarged I_sc_ could be attributed to the faster electron transfer and reduced recombination rate for N-TiO_2_ NRs. These induce the enhanced performance of the solar cells based on N-TiO_2_ NRs.

## Additional file

Additional file 1: Supplementary Material. **Figure S1.** EDS spectrum of 1% N-TiO_2_ NRs. **Figure S2.** Plane-view SEM images of un-doped TiO_2_ (A), and 1% N-TiO_2_ NRs(B). **Figure S3.** Cross-sectional SEM image of perovskite solar cells. (DOCX 826 kb)
