# Supplementary material for: Enhancement of Perovskite Solar Cells Efficiency using N-Doped TiO2 Nanorod Arrays as Electron Transfer Layer
Source: Nanoscale Res Lett. 2017 Jan 17;12:43. doi: 10.1186/s11671-016-1811-0 (PMC5241255; doi:10.1186/s11671-016-1811-0)
Supplement: Supplementary file 1 — Supplementary Material. Figure S1. EDS spectrum of 1% N-TiO2 NRs. Figure S2. Plane-view SEM images of un-doped TiO2 (A), and 1% N-TiO2 NRs(B). Figure S3. Cross-sectional SEM image of perovskite solar cells. (DOCX 826 kb) [file 11671_2016_1811_MOESM1_ESM.docx]

**Supplementary Material**

**Enhancement of Perovskite Solar Cells Efficiency using N-Doped TiO_2_ Nanorod Arrays as Electron Transfer Layer**

Zhen-Long Zhang ^a^, Jun-Feng Li ^a^, Xiao-Li Wang ^a^, Jian-Qiang Qin ^a^, Wen-Jia Shi ^a^, Yue-Feng Liu ^a^, Hui-Ping Gao ^a^, Yan-Li Mao ^a, b, *^

^1^ School of Physics and Electronics, Henan University, Kaifeng 475004, China.

^2^ Institute for Computational Materials Science, Henan University, Kaifeng 475004, China.

**
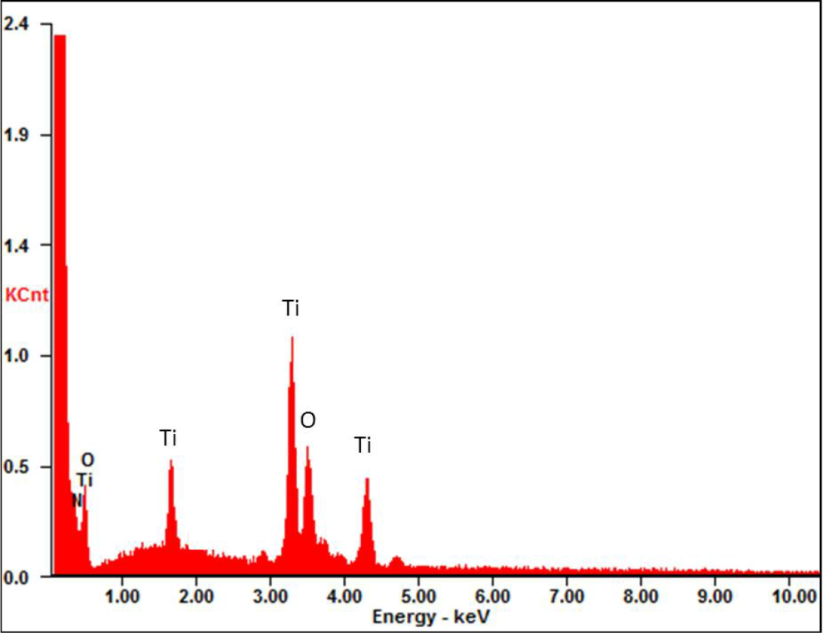
**

Figure S1 EDS spectrum of 1% N-TiO_2_ NRs.

**
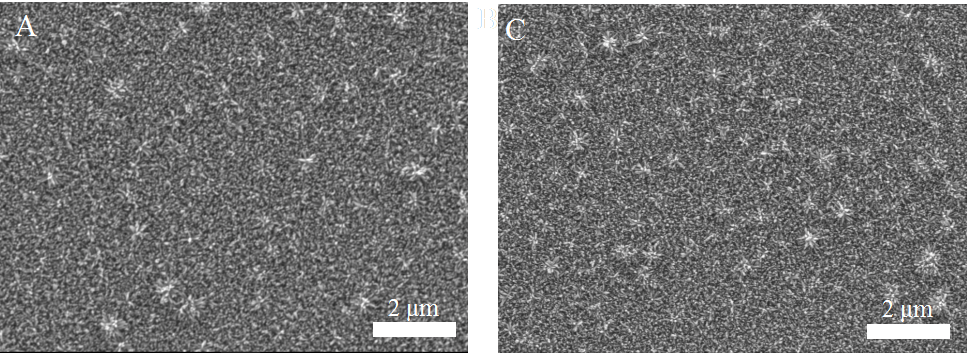
**

Figure S2 Plane-view SEM images of un-doped TiO_2_ (A), and 1% N-TiO_2_ NRs(B).


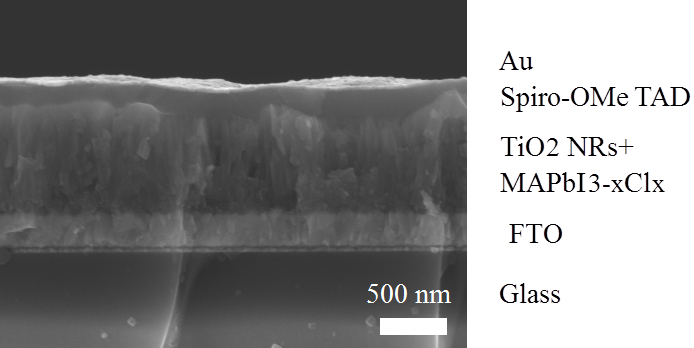


Figure S3 Cross sectional SEM image of perovskite solar cells.
